# Unravelling the secrets of multi-domain lytic polysaccharide monooxygenases (LPMOs)

**DOI:** 10.1107/S2059798323004485

**Published:** 2023-05-30

**Authors:** Gustav Vaaje-Kolstad, Vincent G. H. Eijsink

**Affiliations:** a Faculty of Chemistry, Biotechnology, and Food Science, Norwegian University of Life Sciences (NMBU), PO Box 5003, 1432 Ås, Norway

**Keywords:** multi-domain lytic polysaccharide monooxygenases, copper-dependent enzymes, marine bacteria, *Vibrio* spp., chitin recycling

## Abstract

A new chitin-active AA10 lytic polysaccharide monooxygenase from the marine bacterium *Vibrio campbellii* is described in the paper by Zhou *et al.* [(2023), *Acta Cryst.* D**79**, 479–497].

The discovery of enzymes known as lytic polysaccharide monooxygenases (LPMOs; Vaaje-Kolstad *et al.*, 2005 ▸, 2010 ▸), which catalyze the oxidative cleavage of glycosidic bonds in polysaccharides, has changed our understanding of how nature deals with recalcitrant polysaccharide-rich co-polymeric structures such as plant cell walls. LPMOs have received ample attention in the context of their use for more efficient enzymatic saccharification of chitin- or cellulose-rich biomass (Chylenski *et al.*, 2019 ▸). Interestingly, these abundant enzymes are also encountered in environments where degradation of biomass does not seem pertinent. Furthermore, multiple studies have revealed links between LPMOs and microbial pathogenicity (Vandhana *et al.*, 2022 ▸), for example GbpA in *Vibrio cholerae* (Kirn *et al.*, 2005 ▸; Stauder *et al.*, 2012 ▸; Wong *et al.*, 2012 ▸) and CbpD in *Pseudomonas aeruginosa* (Askarian *et al.*, 2021 ▸).

These latter LPMOs are multi-domain enzymes, starting with a catalytic LPMO domain and ending with a chitin-binding module belonging to family CBM73. In between, they have one or two additional domains with essentially unknown function, the first one of which is called Gbp_A2. The second of these domains resembles the FimC chaperone involved in pili formation in Gram-negative bacteria and is here referred to as Module X. Many carbohydrate-active enzymes, such as cellulases and chitinases, comprise a catalytic domain and one or more substrate-binding domains that are connected by flexible linkers. Crystal structures of such intrinsically flexible multi-domain proteins are very rare.

In this issue, Zhou *et al.* present the crystal structure of a complete four-domain GbpA homologue (Zhou *et al.*, 2023 ▸), *Vh*LPMO10A, produced by *Vibrio campbellii* (also known as *Vibrio harveyi*), an abundant pathogen in marine ecosystems (Zhang *et al.*, 2020 ▸). The previously published structure of GbpA (Wong *et al.*, 2012 ▸) lacks the C-terminal CBM73, whereas the structure of CbpD (Dade *et al.*, 2022 ▸) lacks both Module X (which is absent in this protein) and the CBM73. Important structural features that cannot be easily predicted by state-of-the-art AI algorithms are for example the multimerization state and inter-domain orientations in multi-domain structures. By combining X-ray data with SEC and SAXS data, Zhou *et al.* provide insights in these matters that may give hints to protein function. Based on the data presented, the authors conclude that *Vh*LPMO10A is a monomeric protein with an elongated form, similar to what has been observed for GbpA and CbpD, also using SAXS analysis. Of note, the paper also provides the first crystal structure of a CBM73, for which, so far, only an NMR structure was available (Madland *et al.*, 2021 ▸).

The linkers connecting the domains are rather short (10, 8 and 18 residues, respectively) and contain few glycines (one, zero and two, respectively), suggesting that they are not very flexible, which may have helped in obtaining crystals. The longest linker, connecting the CBM73 to the other three domains, has typical features of an extended linker, with low sequence complexity and abundance of proline (four) and glutamate (four). Indeed, based on various observations, including lacking electron density for the CBM73 in one of the *Vh*LPMO10A monomers in the asymmetric unit, Zhou *et al.* conclude that the CBM moves rather freely and independent of the rest of the protein, as has also been observed in NMR studies of a CBM-containing cellulose-active LPMO (Courtade *et al.*, 2018 ▸). The other three domains may have more fixed orientations relative to each other and their orientation is also similar to that observed for GbpA. Still, for both proteins, SAXS data show a solution structure that is more elongated than the crystal structure, suggesting that the observed relative domain orientations in the crystals are affected by crystal contacts.

Importantly, Zhou *et al.* have gone a long way in the further characterization of their LPMO domain, thus providing important information for the LPMO field. In particular, their data on binding of various metal ions are unique and shed light on the binding potential of the unique copper binding histidine motif in LPMOs. As a point of warning, the pH and temperature optima for ‘LPMO activity’ described in the paper are based on measuring H_2_O_2_ production resulting from an off-pathway oxidase reaction of the LPMO with molecular oxygen (Stepnov & Eijsink, 2023 ▸). While interesting, the catalytic features of the oxidase reaction do not say much about the reaction that leads to oxidation and cleavage of polysaccharide substrates.

Zhou *et al.* suggest that *Vh*LPMO10A might be useful in industrial processing of chitin, which indeed may be the case. However, looking at available functional data for GbpA, it seems likely that proteins such as *Vh*LPMO10A (also?) have a role in the pathogenicity-related physiology of *Vibrio* species. The novel, complete crystal structure of *Vh*LPMO10A and the adjacent functional data provide an excellent starting point for digging deeper into the true biological role of these enzymes. Importantly, GbpA, *Vh*LPMO10A and several other LPMOs with putative roles beyond biomass processing (*e.g.* Askarian *et al.*, 2021 ▸; Zhong *et al.*, 2022 ▸) are all active on chitin, *i.e.* a polymer of a sugar, *N*-acetyl­glucosamine, that is abundant in carbohydrate-containing microbial structures, such as cell walls. It needs to be questioned whether chitin is the true substrate of these LPMOs. In this respect, it is worth noting that the topology of the substrate-binding surfaces of the LPMO domains of GbpA and *Vh*LPMO10A is somewhat different from those of LPMOs known to play a key role in chitin degradation, such as *Sm*LPMO10A (also known as CBP21; see arrows in Fig. 3 in Zhou *et al.*, 2023 ▸).

Despite the structural and functional insights provided by Zhou *et al.*, the biological function of proteins such as *Vh*LPMO10A and GbpA remains largely enigmatic. From work on GbpA, it seems certain that these proteins are important mediators of the interaction between *Vibrio* and relevant host surfaces (Kirn *et al.*, 2005 ▸; Wong *et al.*, 2012 ▸). Indeed, for GbpA it has been shown that the LPMO domain binds to both chitin and intestinal mucin and the CBM73 domain binds to chitin, whereas the combined Gbp_A2 domain and Module X mediate binding of the secreted GbpA protein to the surface of the *Vibrio* cells. Interestingly, the GbpA ortholog of the non-pathogenic bacterium *Aeromonas veronii* Hm21 stimulates epithelial cell proliferation in Zebrafish (Banse *et al.*, 2023 ▸). Most intriguingly, these known biological roles do not explain why these proteins have LPMO activity. Perhaps, degradation of chitin for nutritional and/or invasive purposes is in fact the key role of the LPMO domains. Alternatively, proteins such as *Vh*LPMO10A may have substrates that are yet to be discovered.

## Figures and Tables

**Figure 1:**
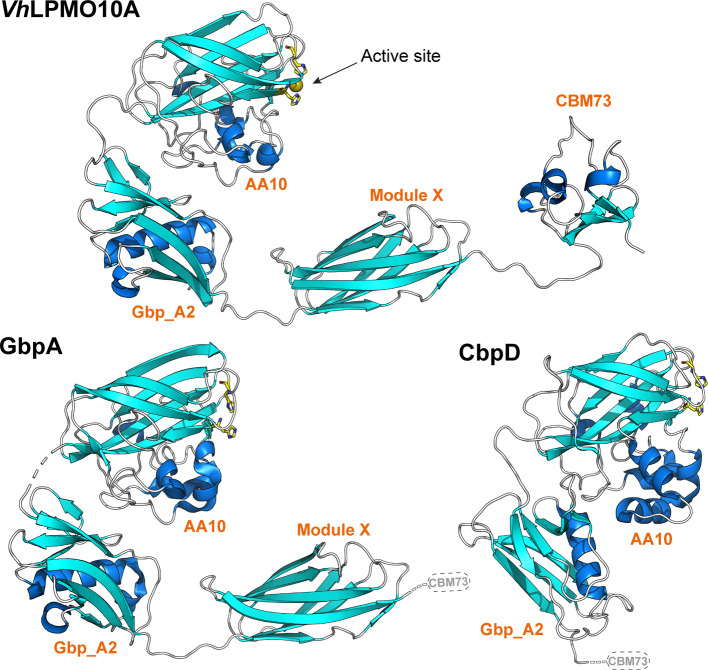
Structure of *Vh*LPMO10A and two similar multi-domain LPMOs. The side chains of the catalytically crucial, copper-binding histidines are shown with yellow carbons and the copper (only shown for *Vh*LPMO10A) appears as an orange sphere. Dashed lines in the structures of GbpA and CbpD indicate structural elements (linker and CBM73 module) that could not be modelled due to protein truncation (GbpA) or insufficient electron density (CbpD).
